# A Practical Treatise on the Disease and Injuries of the Uniary Bladder, Prostrate Gland and Urethra

**Published:** 1851-11

**Authors:** 


ARTICLE III.

*A Practical Treatise on the Diseases and Injuries of the Urinary Bladder, the Prostrate Gland and the Urethra.* By S. D. GROSS, M. D., Prof. of Surgery in the University of Louisville ; Member of the American Medical Association; Author of Elements of Pathological Anatomy, etc., etc., with one hundred and six illustrations. pp. 726 octavo. Blanchard & Lee, Philadelphia, 1851. (From the Publishers; for sale by S. C. Griggs & Co., Chicago.)

This work, for which there was a clear and pressing demand on the part of our Medical Literature, will be greeted by practitioners of Medicine and Surgery with no ordinary welcome, giving as it appears to do copious and clear delineations of the diseases which it discusses and their treatment.

We have not been able yet to give it a perusal, but from the high reputation of the author and the apparent marks of care in its production, we feel no hesitation in advising our readers to procure and study it. For want of time and space we shall be under the necessity of laying it aside for a more extended notice hereafter.

It is got up in the best style of the celebrated publishing house from which it is issued.

